# Retrospective analysis on prognosis of oral cancer patients according to surgical approaches for effective cancer ablation: swing approach versus visor approach

**DOI:** 10.1186/s40902-024-00426-9

**Published:** 2024-04-22

**Authors:** Yun-Ho Kim, Jae-Young Yang, Dong-Min Lee, Jae-Yeol Lee, Dae-Seok Hwang, Mi-Heon Ryu, Uk-Kyu Kim

**Affiliations:** 1 Department of Oral and Maxillofacial Surgery, School of Dentistry, Pusan National University, Yangsan, Republic of Korea; 2 Department of Oral Pathology, School of Dentistry, Pusan National University, Yangsan, Republic of Korea

**Keywords:** Swing approach, Visor approach, Oral cancer, Prognosis

## Abstract

**Background:**

For the surgical treatment of oral cancer, it is sometimes necessary to expand intraoral access within the oral cavity. The “swing approach” that involves lip splitting of the mandible and temporary mandibular osteotomy and the “visor approach” that does not split the lower lip and mandible are mainly used. This study analyzed postoperative outcomes such as complications, recurrence rate, and survival rate by these two approaches. The goal of this study is to evaluate the surgical outcomes of patients using these two approaches, to propose effective perioperative management for oral cancer surgery, and to compare the prognosis of oral cancer patients.

**Materials and methods:**

From 2005 to 2020, 29 patients who underwent surgery at the Department of Oral and Maxillofacial Surgery of Pusan National University Dental Hospital for oral cancer lesions occurred in the mandible, floor of mouth, and tongue were selected for the study. Based on the surgical approach used, a chart review was conducted on various prognostic clinical factors such as the patients’ sex and age, primary site, TNM stage, histopathologic grade, recurrence and metastasis, postoperative survival rate, adjuvant chemo-radiation therapy, satisfaction with aesthetics/function/swallowing, length of hospital stay, tracheostomy and its duration, and neck dissection and its type. Statistical analysis was conducted using SPSS 25.0 (SPSS Inc., Chicago, IL) through Fisher’s exact *t*-test.

**Result:**

There was no statistically significant difference between two groups in terms of clinical and pathological findings, such as survival rate, the need for adjuvant therapies, and the local recurrence rate. Although better outcomes were observed in terms of function, aesthetics, and postoperative complications in the group with visor approach, there was still no statistically significant difference between two groups. However, the duration of hospital stay was shorter in the visor approach group.

**Conclusion:**

There was no statistically significant difference in clinical prognostic factors between the swing approach and the visor approach. Therefore, when choosing between the two approaches for the ablation of oral cancer, it is considered to select the surgical priority approach that can be easy access based on the size and location of the lesion. The visor approach had advantages of aesthetics and healing period.

## Background

The most commonly used approach for intraoral surgery is transoral approach, which is primarily used for the removal of small size tumors or precancerous lesions such as leukoplakia in various parts of the oral cavity [1]. However, for the surgical treatment of more advanced oral cancers, such as mandible involved large tumors or those located at the base of the tongue, it is necessary to perform a resection with an appropriate free surgical margin. But transoral approach often has a limited view, making it difficult to determine the appropriate resection margin, so there is sometimes a need to expand the extent of surgical approach extraorally.

Many extraoral approaches have been developed for efficient resection of advanced oral cancer. The commonly used methods include the swing approach, which involves lip splitting of the mandible, and the visor flap, a modification of the pull-through approach. These two extraoral surgical methods are being applied for the resection of tumors in different parts of the mandible, the tongue, and the floor of mouth. The swing approach, primarily used for the removal of tumors in deep intraoral areas like the tongue base, was first proposed by Roux [2] in 1836 and was introduced in detail by Spiro [3] and others in 1959. It has been considered the main approach for surgeries of tumors in the floor of oral cavity for a long time. This approach involves splitting the lower lip and mandible at the midline to access tumors in the oral cavity floor and tongue, providing a safe and wide access with an acceptable complication ratio [1]. On the other hand, the visor approach does not involve splitting the mandible. Instead, a horizontal incision is made from one mastoid process to the other, following the neck skin crease, and combined with an additional incision in the buccal mucosa and vestibule, the flap is elevated to expose both the oral and extraoral areas. It is mainly used for surgeries in the floor of mouth and the entire mandible and can produce more aesthetically favorable results by avoiding lip splitting [1, 4].

Today, these two surgical approaches are primarily used for the resection of oral cancers, especially in the mandible and floor of mouth. At our institution, the visor flap is primarily considered for exposing lesions suspected of invading the anterior part of the oral floor or the lingual aspect of the mandible as well as for surgery on upper portion of tongue and exposing central anterior parts of the mouth. For surgeries involving deeper locations, such as the posterior part of the mandible including the retromolar area, the posterior or base of the tongue, and in cases of severe patients with TNM stage III or IV, the swing approach, which allows a broader range of access, is preferentially considered. Thus, while the position and size of the lesion are primarily considered, final decisions are made after considering additional factors such as postoperative aesthetic issues, and it is consistent with the other literature [1–4].

The aim of this study is to review the criteria needed for choosing surgical approaches as previously reported, and to evaluate the surgical outcomes of patients who underwent the two approaches for the ablation of oral cancer, especially in the mandible, floor of mouth, and tongue, on clinical prognostic factors, and to identify the pros and cons of each approach for oral cancer surgery. Also, the postoperative outcomes, complications, recurrence rates, and survival rates of patients who underwent each approach were retrospectively analyzed to identify factors affecting the prognosis. Through this, we aimed to suggest appropriate selection criteria for surgical approach, hoping to contribute to improving the surgical outcomes and quality of postoperative life of patients with oral cancer in the mandible, sublingual area.

## Materials and methods

From 2005 to 2020, 29 patients who underwent extraoral surgical approaches for advanced oral cancer lesions in the mandible, floor of mouth, and tongue at the Department of Oral and Maxillofacial Surgery, Pusan National University Dental Hospital, were studied. Patients were classified based on the surgical approach used during the operation, either the swing or visor method. Data on patients’ sex, age, drinking status and history of smoking, primary site, tumor size, histodifferentiation, clinical TNM stage, and pathologic TNM stage were collected. Additionally, locoregional recurrence and 3 to 5 years postoperative survival rate were identified. Retrospective clinical analysis was also conducted on dissatisfaction of function and aesthetics. This was intended to qualitatively evaluate the subjective feelings experienced by the patient themselves, and evaluation items such as pronunciation, mastication, and swallowing were assessed using two categories: “satisfaction” or “dissatisfaction,” employing the term “dissatisfaction” for the evaluation. The occurrence of wound infection and the administration of postoperative adjuvant therapy (chemotherapy, radiotherapy) were also observed. Hospitalization duration, duration for nasogastric tube feeding, and whether tracheostomy was performed and its tube keeping duration were identified and recorded. The data was statistically analyzed for correlations between the clinicopathological factors and the surgical approach using Fisher’s exact *t*-test in SPSS 25.0 (SPSS Inc., Chicago, IL).

## Results

Out of the 29 patients, 19 were male and 10 were female. 16 underwent the swing approach, while 13 underwent the visor approach. For clinicopathological factors, the 29 patients were categorized based on sex, age, alcohol consumption and smoking status, primary site, tumor size, clinical/pathological/histological stage, presence of lymph node and distant metastasis, type of flap used for reconstruction, and duration of hospitalization. The results were summarized for these detailed clinical and pathological evaluation factors (Table 1, *: *P* < 0.05).

**Table 1:** Clinicopathologic characteristics of 29 patients

		Swing approach (*n* = 16)	Visor approach (*n* = 13)	*P*-value*
Sex	Male	11 (68.8%)	8 (61.5%)	0.7141
	Female	5 (31.2%)	5 (38.5%)	
Age (Mean: 57.8)	> 60	11 (68.8%)	8 (61.5%)	0.7141
	≤ 60	5 (31.2%)	5 (38.5%)	
	Mean	54.6	61.7	
Drinking	O	6 (37.5%)	6 (46.2%)	0.7163
	X	10 (62.5%)	7 (53.8%)	
Smoking	O	4 (25.0%)	3 (23.1%)	1.0000
	X	12 (75.0%)	10 (76.9%)	
Primary site	Mandible	9 (56.2%)	8 (61.5%)	1.0000
	FOM*, tongue	7 (43.8%)	5 (38.5%)	
Tumor size	T1-T2	3 (18.8%)	7 (53.8%)	0.0641
	T3-T4	13 (81.2%)	6 (46.2%)	
Clinical stage	I, II	1 (6.2%)	2 (15.4%)	0.5731
	III, IV	15 (93.8%)	11 (84.6%)	
Pathologic stage	I, II	2 (12.5%)	4 (30.8%)	0.3640
	III,IV	14 (87.5%)	9 (69.2%)	
Histopathologic grade	Well-differentiated	12 (75.0%)	2 (15.4%)	*0.0025
	Moderately differentiated	4 (25.0%)	11 (84.6%)	
Flap used for reconstruction	RFFF*	5 (31.2%)	9 (69.2%)	0.3647
	FFF*	9 (56.3%)	4 (30.8%)	
	ALT FF*	2 (12.5%)	(-)	
Neck dissection	SOHND*	13 (81.3%)	10 (76.9%)	1.0000
	mRND*	3 (18.7%)	3 (23.1%)	

^*^FOM floor of mouth, *RFFF* radial forearm free flap, *FFF* fibula free flap, *ALT FF* antero-lateral thigh free flap, *SOHND* supra-omohyoid neck dissection, *mRND* modified radical neck dissection

*P*-value*: by Fisher’s exact *t*-test

The only factor that showed a statistically significant difference between the two approach methods was the histopathologic grade, a histopathological analysis of tumor mass excised by surgery. Of the 16 cases that underwent by the swing approach, 12 were histologically well-differentiated. In contrast, in the visor approach, the majority, 11 out of 13 cases, were moderately differentiated. No statistically significant differences were observed in other factors.

For postoperative prognosis factors, locoregional recurrence, postoperative 3-year and 5-year survival status, and the presence or absence of postoperative adjuvant therapy were analyzed in relation to postoperative prognosis (Table 2, *: *P* < 0.05).

**Table 2:** Postoperative prognosis factors and adjuvant therapy

		Swing approach (*n* = 16)	Visor approach (*n* = 13)	*P*-value*
Locoregional recurrence	O	5 (31.3%)	1 (7.7%)	0.1834
	X	11 (68.7%)	12 (92.3%)	
Cervical nodal metastasis	O	6 (37.5%)	8 (61.5%)	0.2723
	X	10 (62.5%)	5 (38.5%)	
Distant metastasis	O	2 (12.5%)	3 (23.1%)	0.6322
	X	14 (87.5%)	10 (76.9%)	
Postoperative 3-year survival	O	13 (81.3%)	10 (76.9%)	1.0000
	X	3 (18.7%)	3 (23.1%)	
Postoperative 5-year survival	O	10 (83.3%)	2 (66.7%)	0.5165
	X	2 (16.7%) (except 4 cases within 5 years)	1 (33.3%) (except 10 cases within 5 years)	
Postoperative adjuvant therapy	O	8 (50.0%)	11 (84.6%)	0.1142
	X	8 (50.0%)	2 (15.4%)	

*P*-value*: by Fisher’s exact *t*-test

The visor flap showed a lower recurrence rate, but the survival rate was higher for the swing approach. The need for postoperative adjuvant therapy was higher in the visor flap, but no statistically significant difference was observed. The subjects of this study were patients who underwent surgery up to 2020, and data collection was conducted in 2022. Therefore, for patients who had surgery after 2018, the 5-year survival rate was calculated as a 3-year survival rate instead.

For postoperative complication factors, the results of the analysis on postoperative functional dissatisfaction, such as aesthetics, speech, and mastication, the occurrence of wound infection, the implementation of neck dissection, and the type of reconstructive flap used were presented (Table 3, *: *P* < 0.05).

**Table 3:** Postoperative complication factors

			Swing approach (*n* = 16)	Visor approach (*n* = 13)	*P*-value*
Aesthetic dissatisfaction		O	2 (12.5%)	1 (7.7%)	1.0000
		X	14 (87.5%)	12 (92.3%)	
Functional dissatisfaction	Speech	O	2 (12.5%)	1 (7.7%)	1.0000
		X	14 (87.5%)	12 (92.3%)	
	Mastication	O	5 (31.2%)	2 (15.4%)	0.4100
		X	11 (68.8%)	11 (84.6%)	
	Swallowing	O	2 (12.5%)	1 (7.7%)	1.0000
		X	14 (87.5%)	12 (92.3%)	
Wound infection		O	4 (25.0%)	1 (7.7%)	0.3432
		X	12 (75.0%)	12 (92.3%)	

*P*-value*: by Fisher’s exact *t*-test

The visor flap showed fewer aesthetic and functional dissatisfactions, and there were also fewer cases of wound infection. However, these differences were not statistically significant.

For treatment-related factors, the results of the analysis on the duration of hospitalization, the need for blood transfusion, the number of days with bed rest, the number of days the nasogastric tube was maintained, and whether tracheostomy was performed and its duration were recorded (Table 4, *: *P* < 0.05).

**Table 4:** Treatment-related factors

		Swing approach (*n* = 16)	Visor approach (*n* = 13)	*P*-value*
Length of hospital days (mean: 46.6) (SD*: 36.5)	≥ 30 days	13 (81.3%)	5 (38.5%)	*0.0266
	< 30 days	3 (18.7%)	8 (61.5%)	
	Mean	51.0	43.3	
	SD*	20.7	49.2	
	≥ 46 days	8 (50.0%)	2 (15.4%)	0.1142
	< 46 days	8 (50.0%)	11 (84.6%)	
Blood transfusion	O	2 (12.5%)	2 (15.4%)	1.0000
	X	14 (87.5%)	11 (84.6%)	
Length of bed rest (walking restrictions) (mean: 14.1) (SD*: 6.3)	≥ 14 days	9 (56.2%)	5 (38.5%)	0.4621
	< 14 days	7 (43.8%)	8 (61.5%)	
	Mean	15.2	12.9	
	SD*	6.9	5.3	
Days with nasogastric feeding (mean: 24.0) (SD*: 14.9)	≥ 24 days	7 (43.8%)	4 (30.8%)	0.7021
	< 24 days	9 (56.2%)	9 (69.2%)	
	Mean	22.6	26.1	
	SD*	9.2	19.5	
Tracheostomy	O	5 (31.3%)	3 (23.1%)	0.6968
	X	11 (68.7%)	10 (76.9%)	
Days with tracheostomy tube (mean: 16.2) (SD*: 4.7)		(*n* = 5)	(*n* = 3)	1.0000
	≥ 16 days	3 (60.0%)	1 (33.3%)	
	< 16 days	2 (40.0%)	2 (66.7%)	
	Mean	15.6	17.3	
	SD*	5.0	4.0	

*P*-value*: by Fisher’s exact *t*-test, SD*: standard deviation

Comparison of the mean values between two groups was conducted. The duration of hospitalization was significantly shorter for the visor approach, and there were also far fewer cases of extended hospital stays. At our institution, for patients undergoing severe oral cancer surgery, 2 weeks of post-surgery healing period is allocated for the surgical sites. And, to check the overall health recovery, including mouth opening, eating, and walking, an additional 2 weeks of hospital stay is added, making a total of about 30 days as the basic hospitalization period. Then, depending on the patient’s condition, the duration of the hospital stay may be extended or reduced as part of patient care. Based on the above criteria, approximately 30-day of hospitalization period was used as an indicator for assessing the degree of the patient’s health recovery.

The period of bed rest was also shorter for the visor flap, but the duration of maintaining the L-tube (nasogastric feeding) was actually longer. The rate of tracheostomy procedures was slightly higher during the swing approach. Most perioperative care factors did not show a statistically significant difference, with the sole exception being the duration of hospitalization, which was statistically significantly shorter for the visor approach.

**Fig. 1:**
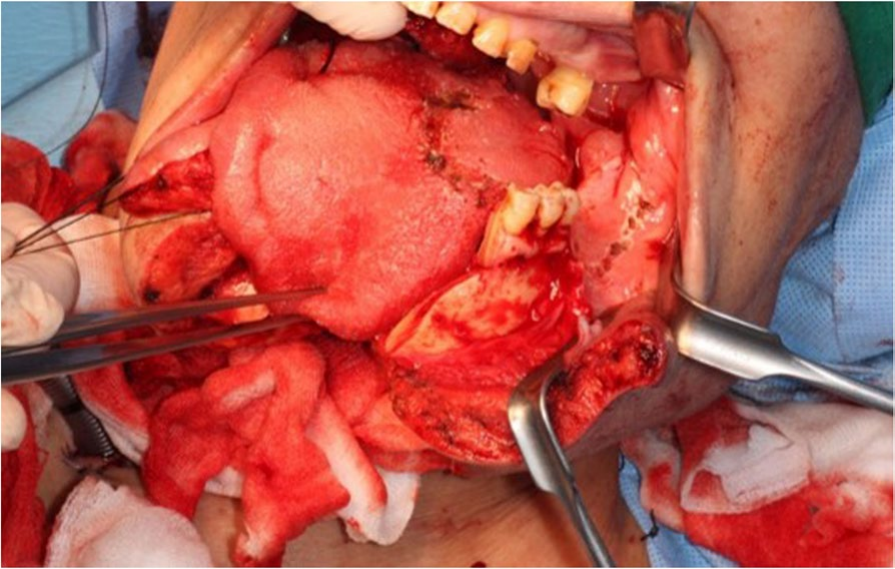
Swing approach for excision of tongue base cancer

**Fig. 2:**
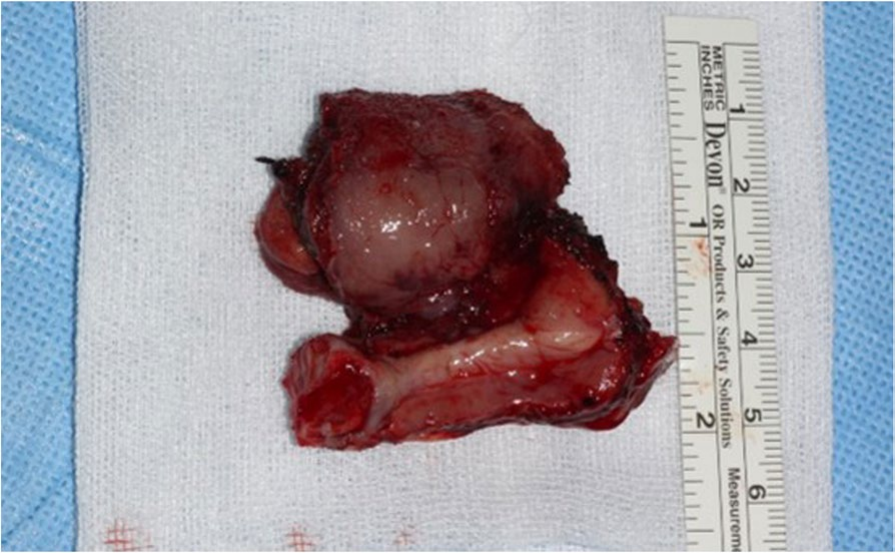
Excised tongue cancer and adjacent tissue lesions

**Fig. 3:**
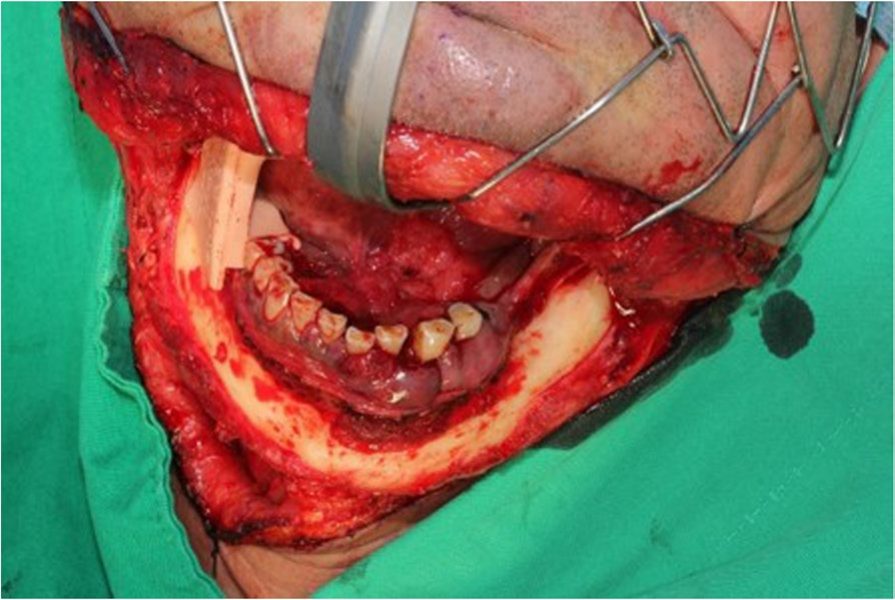
Visor approach for excision of floor of mouth and mandibular cancer lesion

**Fig. 4:**
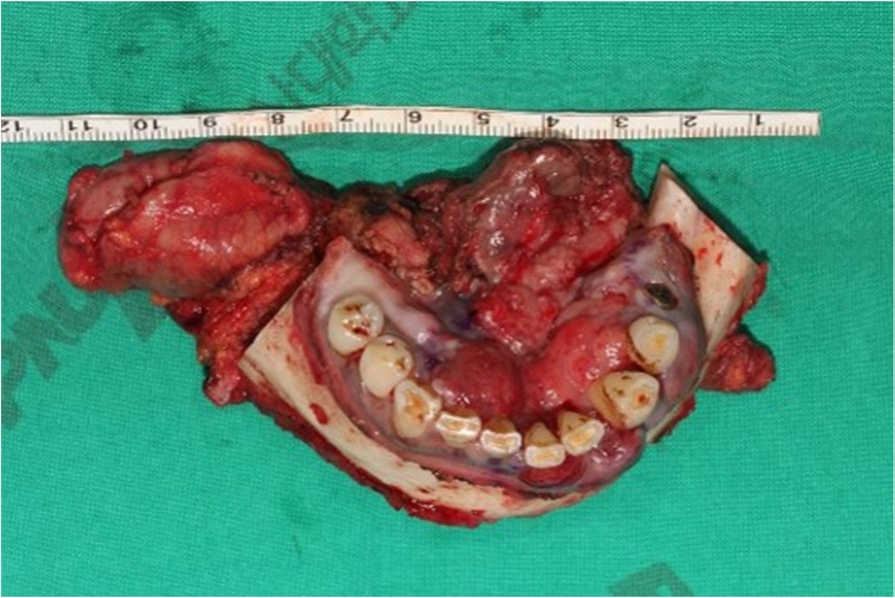
Excised floor of mouth and mandibular cancer lesion

Examples were provided of our cases where the two surgical methods were applied for the resection of oral cancers that occurred in different areas (swing approach—Figs. 1 and 2; visor approach—Figs. 3 and 4).

## Discussion

There have not been many comparative studies on which of the two surgical approach methods, the swing approach, which accesses through lip splitting and mandibulotomy, and the visor approach, which accesses by making an incision in the neck skin without lip splitting, is more suitable for cancer ablation. However, when actually performing surgeries for oral cancer ablation, whether to use the swing method, which involves dividing the mandible for access, or the visor method, there is a need for the surgeon to make a careful decision based on solid evidence. Surgeons should refer to existing literature for the theoretical background of the decision on the surgical approach, and additionally, a new clinical study should be continuously conducted to provide clearer criteria for determining the surgical approach method. In this study, to compare the surgical outcomes of the two approaches, retrospective analysis of oral cancer surgery in the mandible, tongue, and oral floor areas was divided into four major fields: clinicopathological factors, postoperative prognosis factors, postoperative complication factors, and treatment-related factors.

In the clinicopathological factors section, detailed items related to clinical and pathological evaluations, such as sex and age, drinking and smoking status, primary site, tumor size, and clinical/pathological/histological stages, were assessed. The average age of the patient group that underwent the visor approach was 61.7 years, which was higher than the average of 54.6 years for the swing approach group, but this was not statistically significant. Among the 16 cases of the swing approach, 13 cases had larger tumors, around T3–T4, while in the visor approach group, 7 out of 13 cases were T2 or lower. Although this was not statistically significant, with a *P*-value of 0.0641, which is close to 0.05. It is considered necessary to conduct studies with a larger number of clinical cases to derive statistically significant results.

The histopathologic grade was the only item in the clinicopathological factors that showed a statistically significant difference between the two approaches. However, since this result can only be known after the surgery, it would be impractical to use it as a criterion for deciding the approach before surgery. In a study by Benjamin et al. [5], it was stated that there was no significant difference between the two approaches in terms of pathological margins and local recurrence rates.

In one another study, it was suggested that the swing approach, which involves directly opening and accessing the mandible, might be superior in terms of the surgical field of view [4]. Additionally, Nam W [1] and Tae K [4] stated that while the visor approach is suitable for accessing the anterior and central parts of the mandible and oral cavity, it is not ideal for accessing the posterior oral cavity and the area around the molars. Therefore, it may be necessary to consider whether different approaches should be applied depending on the primary site. In this study, among the 16 cases using the swing approach, 9 were in the mandible, and 7 were in the oral floor and tongue. For the 13 cases using the visor approach, 8 were in the mandible, and 5 were in the oral floor and tongue. Overall, the ratios were similar, indicating that the location of the primary site itself did not significantly influence the surgeon’s choice of approach. Since no significant differences were observed in postoperative prognosis between the two methods, it was thought that rather than deciding the approach solely based on the primary site, it would be more appropriate to make a decision considering the overall location and size of the lesion, and other factors for each case.

For the postoperative prognosis factors, evaluations were made on locoregional recurrence, cervical node/distant metastasis, survival status over 3 and 5 years post-surgery, and the necessity of postoperative adjuvant therapies such as radiation and chemotherapy. It was observed that the survival rate over 3 and 5 years was higher for the swing approach, but there was also a higher incidence of locoregional recurrence. In the visor group, the proportion of patients who received postoperative adjuvant therapies such as radiation and chemotherapy was much higher, though not statistically significant. But the use of the visor approach alone was not the simple reason for the higher incidence of postoperative adjuvant therapy in these clinical research cases. Instead, it was a comprehensive judgment based on the location and size of the lesion at the time of surgery as well as the postoperative pathological report of the resected specimen.

In the most recent study comparing the two approaches by Vyshnavi [6], it was noted that the surgery duration was longer in the visor group, but the adequacy of exposure, safety margins, surgical outcomes related to healing, and complications were similar in both groups. Additionally, in 2018, Leslie E. Cohen et al. [7] mentioned that there were no significant differences observed in surgical site complications or surgery duration between the two approaches, and both methods can be seen as effective for tumor removal.

In the postoperative complication factors category, evaluations were made on dissatisfaction related to aesthetics, speech, mastication (chewing), and swallowing. Additionally, the occurrence of wound infection was also assessed. In 2023, Hardingham et al. [8] mentioned that difficulty in swallowing is a common outcome after surgical resection of oral or oropharyngeal squamous cell carcinoma. Especially during the visor approach, while there are aesthetic advantages, there is a potential to induce sensory abnormalities in the mental region [9]. In this study, during the postoperative complication assessment, the focus was on aesthetic aspects, and an evaluation of sensory abnormalities was not conducted. During the postoperative assessment of patients, the sensory abnormality status was not recorded in medical records for all patients; hence, this item could not be included in this study. Li et al. [10] proposed a modified visor approach to address these sensory abnormalities by preserving the mental nerve, and as a result, they stated that lip splitting and mandibulotomy were unnecessary. In Li’s study, complications related to swallowing, mastication, and speech also showed similar results between the two approaches [10].

There have been numerous reports of complications related to the mandibulotomy performed during the swing approach. In a study by Byun et al. [11] on complications and contributing factors of mandibulotomy in 2000, postoperative nonunion at the mandibulotomy site was reported. This was attributed to the instability caused by the movement between bone fragments when intraosseous wiring was used. However, it was mentioned that initial nonunion did not occur when miniplates or plates were used for fracture fixation. Most of the other complications were related to postoperative radiation therapy at the mandibulotomy site. McCann et al. [12] reported nonunion and radiation-induced osteonecrosis in patients who received preoperative radiation therapy. Altman and Bailey [13] reported nonunion in the mandible of patients who underwent radiation therapy and recommended avoiding mandibulotomy in patients who received radiation therapy to the mandible. However, Shah et al. [14] stated that pre/postoperative radiation therapy does not affect bone healing. Davidson et al. [15, 16] also mentioned that preoperative radiation therapy does not influence the occurrence of nonunion or malunion related to mandibulotomy, and mandibulotomy can be used during the swing approach without an increased incidence of radiation-induced osteonecrosis, regardless of radiation therapy.

In Vyshnavi’s study [6], the most common complication in both groups was found to be the orocutaneous fistula, indicating wound infection. In a study by Cliento BW et al. [5], it was mentioned that most of the patients who developed fistulas had undergone postoperative radiation therapy. In this study, wound infection occurred in 4 cases with the swing approach, of which 2 had undergone postoperative adjuvant therapy, and in the 1 case with the visor approach, postoperative adjuvant therapy was also used. Although statistical analysis is difficult due to the small sample size, it can be inferred that there may be some infections correlation with postoperative radiation therapy depending on the surgical approach.

There was no clear evidence regarding the induction of postoperative complications by anticancer chemotherapy. Byun et al. [11] reported that complications occurred in 16% of patients who did not receive chemotherapy after oral cancer surgery and 60% of those who did. However, since there were no patients who underwent only chemotherapy, it was reported that an accurate assessment was difficult. In this study, all patients who received chemotherapy also underwent radiation therapy, making it difficult to determine the sole side effect of chemotherapy, suggesting the need for further research.

In this study, the swing approach showed slightly more aesthetic and functional dissatisfaction, and the occurrence of wound infections was also higher. However, this was not statistically significant. In a study comparing the two approaches in functional and aesthetic areas by Leslie E. Cohen et al. in 2018 [7], it was found that the visor flap method showed improvements in both functional and aesthetic aspects. It was also noted that patients who underwent the swing approach had worse outcomes in terms of eating and language functions compared to those who underwent the visor approach. This is presumed to be due to lip splitting, suggesting that the visor approach might offer slight advantages in mastication and speech. Overall, in terms of postoperative functional aspects such as mastication, swallowing, and speech, it is believed that the visor approach can achieve at least similar or slightly better results than the swing approach.

In the treatment-related factors section, analyses were conducted on the duration of hospitalization, the necessity of blood transfusion, the number of days of bed rest, the duration of nasogastric tube maintenance, and the necessity and duration of tracheostomy. In terms of the average hospitalization period, the visor approach required a shorter stay of 43.3 days compared to the 51.0 days for the swing approach, which showed a statistically significant difference. Additionally, while not statistically significant, the duration of nasogastric feeding and tracheostomy maintenance appeared to be longer in the swing approach. This is believed to be a result of a more invasive approach applied to the upper digestive organs and trachea responsible for swallowing when performing lip splitting during surgery. In a study by Devine JC et al. in 2001 [17], it was mentioned that during the swing approach, the genial muscle attached to the center of the mandible needs to be detached, which could delay the postoperative recovery of mastication and swallowing abilities, and this was considered the main reason.

The duration of nasogastric tube maintenance and the number of days of bed rest were clinical factors specifically analyzed in this study to compare the differences between the two surgical approaches, and it was difficult to find similar related studies. However, in terms of the duration of hospitalization, similar results to this study could be found. In Li’s study [10], it was mentioned that without performing lip splitting and mandibulotomy, both aesthetic results and the duration of hospitalization could be reduced in visor approach.

In this study, despite conducting comparative analysis across many evaluation items, it was disappointing that there were few items showing statistically significant differences in results. Despite objective reference points that could be considered indications for choosing a surgical approach were not clearly identified, this research is deemed valuable as it provided various clinical and pathological factors for comparison and analysis, so that surgeons can predict and refer to patient prognosis patterns after applying the two surgical methods. It is expected that future continuous clinical research, collecting and analyzing more than 30 cases per surgical approach, will yield statistically significant results, and this would provide more reliable and meaningful criteria for applying surgical methods. Further analysis through a secondary clinical study is intended.

## Conclusion

When comparing the two surgical approaches for oral cancer resection, no statistically significant difference was observed in the postoperative therapeutic prognosis of oral cancer between the two methods. Therefore, when choosing between the two approaches for resecting oral cancer, it is essential to prioritize the method that allows easier access to the lesion based on its size and location. The visor approach had aesthetic advantages and also reduced the patient’s hospitalization duration. The results of this study provide important indicators for choosing a more efficient approach for oral cancer ablative surgery, especially for extraoral access to the mandible, floor of mouth, and tongue, and are expected to contribute to improving patient outcomes.

## Data Availability

All data generated or analyzed during this study are included in this published article.

## References

[1] Nam W (2010). Principles and practice of oral cancer surgery. J Korean Dent Assoc.

[2] Butlin HT (1885). Diseases of the tongue. Clinical Manuals for Practitioners and Students of Medicine.

[3] Dubner S, Spiro RH (1991). Median mandibulotomy: a critical assessment. Head Neck.

[4] Tae K (2009). Surgical management of oral cancer. Hanyang Med Rev.

[5] Cilento BW, Izzard M, Weymuller EA, Futran N (2007). Comparison of approaches for oral cavity cancer resection: lip-split versus visor flap. Otolaryngol Head Neck Surg..

[6] Vyshnavi V, Azeem Mohiyuddin SM, Mohammadi K (2023). Comparison of visor access approach with lower lip split approach in resection of oral cancers. Indian J Otolaryngol Head Neck Surg.

[7] Cohen LE, Morrison KA, Taylor E, Jin J, Spector JA, Caruana S, Rohde CH (2018). Functional and aesthetic outcomes in free flap reconstruction of intraoral defects with lip-split versus non–lip-split incisions. Ann Plast Surg.

[8] Hardingham NM, Ward EC, Clayton NA, Gallagher RA (2023). Does the mandibular lingual release approach impact post-operative swallowing in patients with oral cavity and/or oropharyngeal squamous cell carcinomas: a scoping review. Speech Lang Hear.

[9] LaFerriere KA, Sessions DG, Thawley SE, Wood BG, Ogura JH (1980). Composite resection and reconstruction for oral cavity and oropharynx cancer: a functional approach. Arch Otolaryngol.

[10] Li W, Li R, Safdar J, Huang S, Xu Z, Tan X, Sun C (2014). Modified visor approach applied to total or subtotal glossectomy and reconstruction: avoidance of lip splitting and mandibulotomy and cutting off mental nerve. Tumor Biol.

[11] Byun SK, Choi EC, Park WS, Lee EW, Cha IH (2000). Mandibulotomy, a surgical approach for oral cancer: its complications and contributing factors. Kor Oral Maxillofac Surg.

[12] McCann KJ, Irish JC, Gullane PJ, Holmes H, Brown DH, Rotstein L (1994). Complications associated with rigid fixation of mandibulotomies. J Otolaryngol.

[13] Altman K, Bailey BMW (1996). Non-union of mandibulotomy sites following irradiation for squamous cell carcinoma of the oral cavity. Br J Oral Maxillofac Surg.

[14] Shah JP, Kumaraswamy SV, Kulkarni V (1993). Comparative evaluation of fixation methods after mandibulotomy for oropharyngeal tumors. Am J Surg.

[15] Davidson J, Freeman J, Gullane P, Rotstein L, Birt D (1988). Mandibulotomy and radical radiotherapy: compatible or not?. J of Otolaryngology.

[16] Davidson J, Freeman J, Birt D (1989). Mandibulotomy in the irradiated patient. Arch Otolaryngol Head Neck Surg.

[17] Devine JC, Rogers SN, McNally D, Brown JS, Vaughan ED (2001). A comparison of aesthetic, functional and patient subjective outcomes following lip-split mandibulotomy and mandibular lingual releasing access procedures. Int J Oral Maxillofac Surg.

